# Supplementary material for: Gut microbial bile and amino acid metabolism associate with peanut oral immunotherapy failure
Source: Nat Commun. 2025 Jul 9;16:6330. doi: 10.1038/s41467-025-61161-x (PMC12241578; doi:10.1038/s41467-025-61161-x)
Supplement: Supplementary file 1 — Supplementary Information [file 41467_2025_61161_MOESM1_ESM.pdf]

Supplementary Data

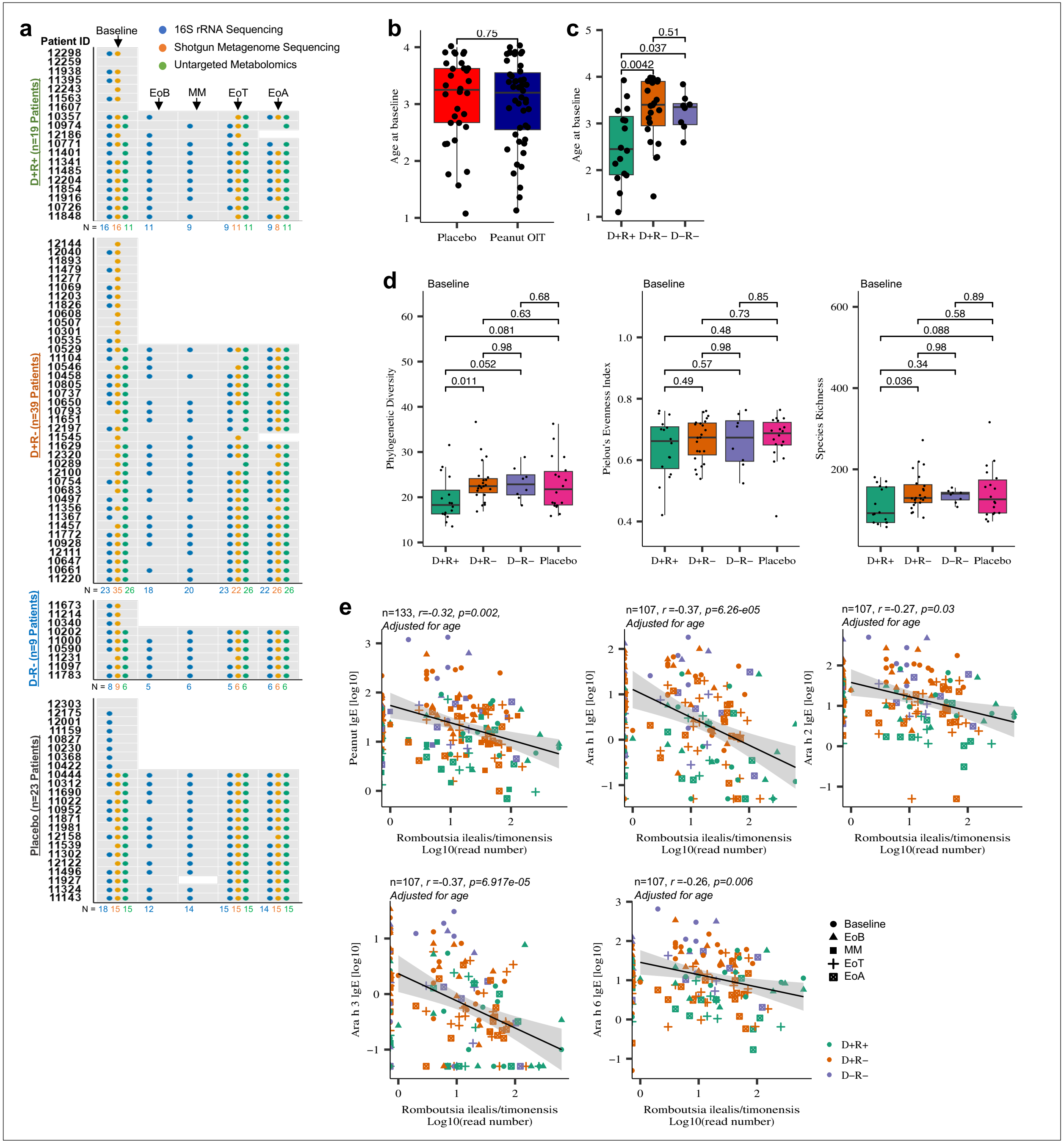

Supplementary Fig. 1. a, Longitudinal collection and multi-omics profiling of fecal samples from 90 participants who completed the POIT or placebo protocols (per protocol group) in the IMPACT trial. Each gray rectangular cell represents a fecal sample collected at one of five time points: baseline, end of build-up (EoB), mid-maintenance (MM), end of treatment (EoT), and end of avoidance (EoA). Samples are stratified by treatment outcome: D+R+ (desensitization and remission), D+R− (desensitization without remission), D−R− (no desensitization or remission), and placebo (shown separately). Numbers below each panel indicate the number of fecal samples processed for 16S rRNA sequencing (blue), shotgun metagenomics (orange), and untargeted

metabolomics (green). The number of patients in each group is indicated on the y-axis (*n*=19 for D+R+, 39 for D+R−, 9 for D−R−, and 23 for placebo). Of 327 collected fecal samples, 266 from 81 participants passed quality control and were included in 16S rRNA analysis. Shotgun metagenomic data were available for 184 samples from 80 participants at three time points: baseline, EoT, and EoA. Eight placebo participants (bottom left of the placebo group) were excluded from metagenomic analysis due to incomplete sampling at EoT and EoA (see Methods). **b**, Age did not differ significantly at baseline between the placebo (*n*=18) and POIT (*n*=47) arms (two-sided ANOVA, *P*=0.75). **c**, Participants who achieved POIT-induced remission (D+R+, *n*=16) were significantly younger than those in the D+R− (*n*=23, *P*=0.0042) and D−R− (*n=8*, *P*=0.037) groups (two-sided ANOVA), consistent with the original study. **d**, Baseline fecal bacterial α-diversity metrics—Faith's phylogenetic diversity, Pielou's evenness, and Chao1 richness—across outcome groups and placebo (n=*16* for D+R+, *n=23* for D+R−, *n=8* for D−R−, and *n=18* for placebo). Two-sided Wilcoxon rank-sum test. **e**, Abundance of *Romboutsia ilealis/timonensis* negatively correlates with all measured peanut-specific IgE (*n=133*, *r*=−0.32, *P=0.002*) and component-specific IgE levels: Ara h 1 (*n=107*, *r=−0.37, P=6.26×10⁻⁵),* Ara h 2 (*r*=−0.27, *P*=0.03), Ara h 3 (r=*−0.37,* P=*6.92×10⁻⁵*), and Ara h 6 (*r*=−0.26, *P*=0.006). Two-sided Pearson correlations adjusted for age. *LME*: linear mixed-effects model. Boxplots (**b–d**) show the median (center line), 25th and 75th percentiles (box bounds), and whiskers extending to 1.5× the interquartile range. Source data are provided in the Source Data file.

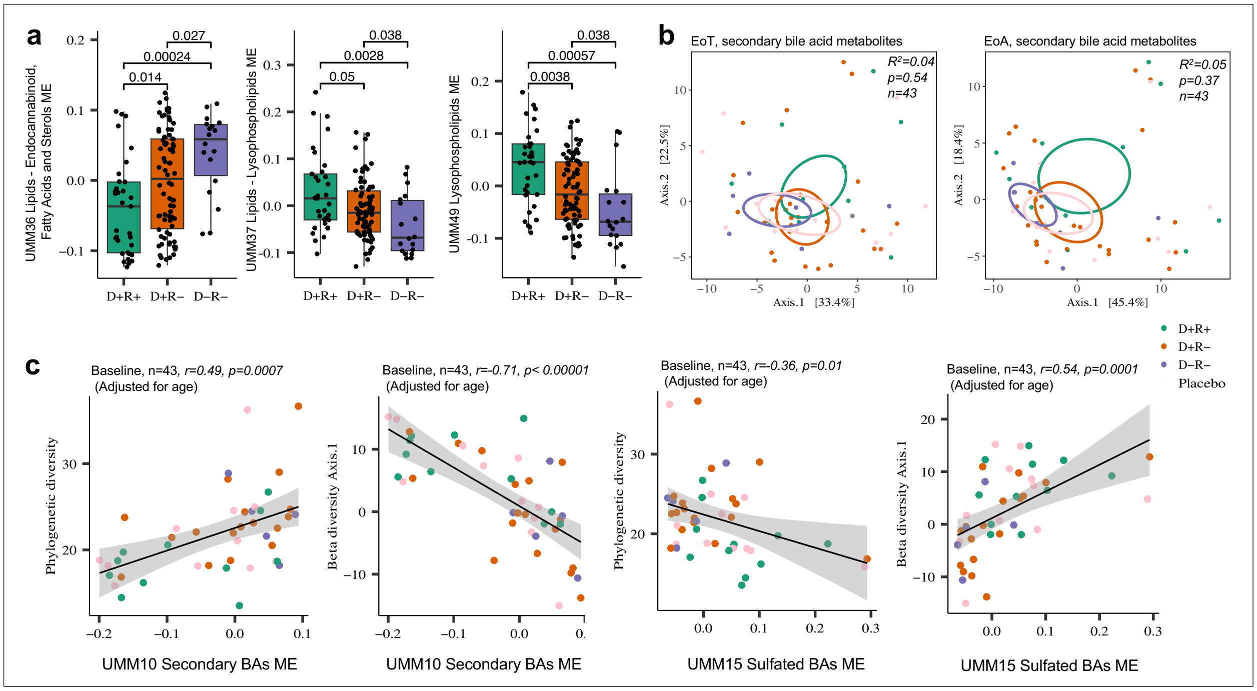

Supplementary Figure 2. a, Differences in module eigengenes of UMM36, UMM37, and UMM49 amino acid modules between POIT outcome groups. Boxplots show the median (center line), 25th and 75th percentiles (box bounds), and whiskers extend to 1.5× the interquartile range. Statistical comparisons were performed using two-sided Wilcoxon rank-sum tests (*n*=129; D+R+=33, D+R−=78, D−R−=18). **b,** Fecal bile acid (BA) metabolite composition does not differ between POIT outcome groups at end of treatment (EoT; R^2=0.04, *P*=0.54, *n*=43) or end of avoidance (EoA; R^2=0.05, *P*=0.37, *n*=43). Shown are ordinations of EoT and EoA secondary BA metabolites; PERMANOVA analyses were performed using the Euclidean dissimilarity metric. **c,** Bile acid modules UMM10 and UMM15, which are significantly associated with POIT outcome, correlate

with phylogenetic diversity and gut microbiome composition (P<0.05; two-sided Pearson correlations, adjusted for age).Source data are provided in the Source Data file

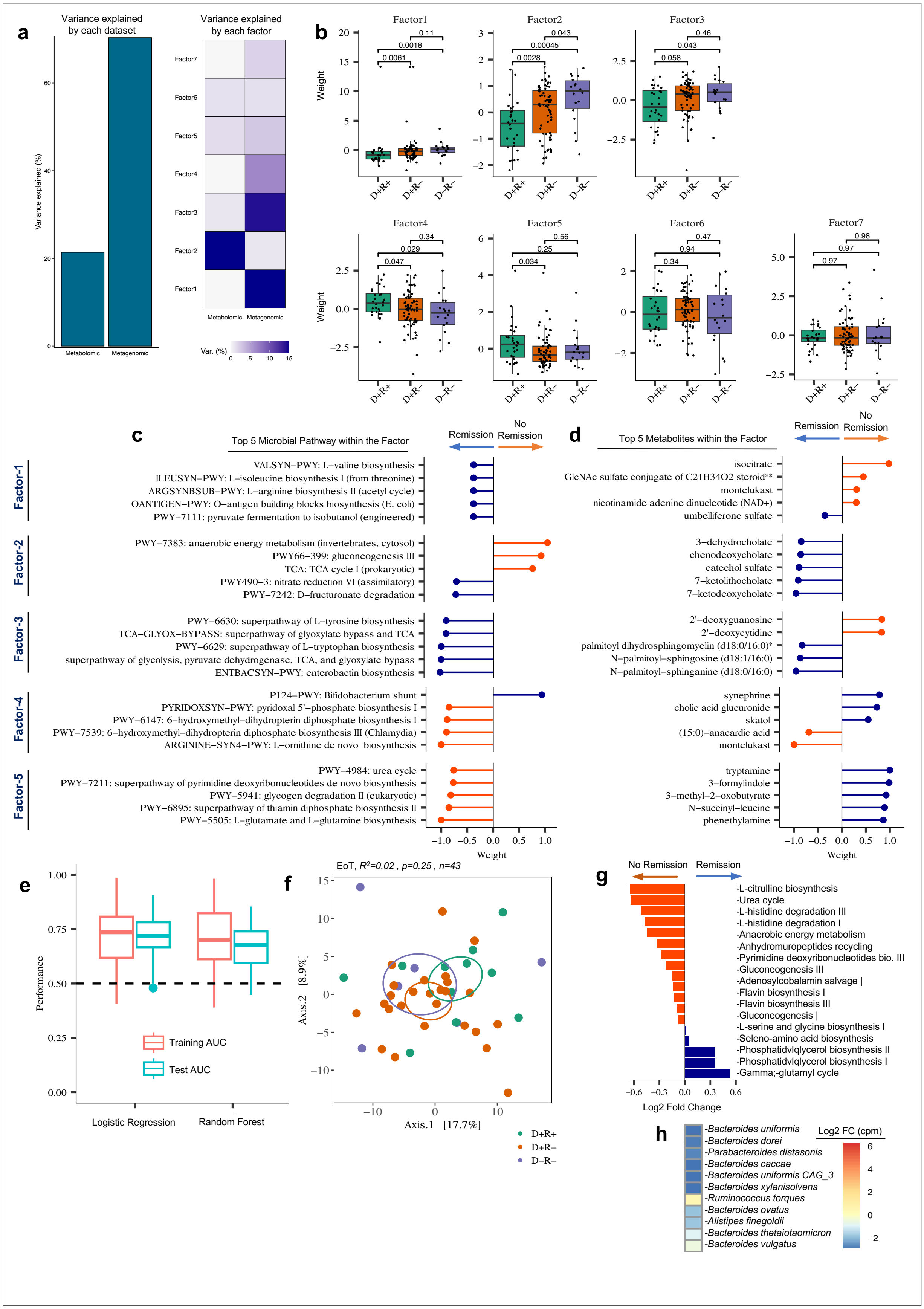

a
Variance explained by each dataset
Variance explained (%)
Metabolomic
Metagenomic
Variance explained by each factor
Factor7
Factor6
Factor5
Factor4
Factor3
Factor2
Factor1
Var. (%)
b
Factor1
Factor2
Factor3
Factor4
Factor5
Factor6
Factor7
Weight
D+R+
D+R−
D−R−
c
Top 5 Microbial Pathway within the Factor
Remission
No Remission
Factor-1
VALSYN−PWY: L−valine biosynthesis
ILEUSYN−PWY: L−isoleucine biosynthesis I (from threonine)
ARGSYNBSUB−PWY: L−arginine biosynthesis II (acetyl cycle)
OANTIGEN−PWY: O−antigen building blocks biosynthesis (E. coli)
PWY−7111: pyruvate fermentation to isobutanol (engineered)
Factor-2
PWY−7383: anaerobic energy metabolism (invertebrates, cytosol)
PWY66−399: gluconeogenesis III
TCA: TCA cycle I (prokaryotic)
PWY490−3: nitrate reduction VI (assimilatory)
PWY−7242: D−fructuronate degradation
Factor-3
PWY−6630: superpathway of L−tyrosine biosynthesis
TCA−GLYOX−BYPASS: superpathway of glyoxylate bypass and TCA
PWY−6629: superpathway of L−tryptophan biosynthesis
superpathway of glycolysis, pyruvate dehydrogenase, TCA, and glyoxylate bypass
ENTBACSYN−PWY: enterobactin biosynthesis
Factor-4
P124−PWY: Bifidobacterium shunt
PYRIDOXSYN−PWY: pyridoxal 5'−phosphate biosynthesis I
PWY−6147: 6−hydroxymethyl−dihydropterin diphosphate biosynthesis I
PWY−7539: 6−hydroxymethyl−dihydropterin diphosphate biosynthesis III (Chlamydia)
ARGININE−SYN4−PWY: L−ornithine de novo biosynthesis
Factor-5
PWY−4984: urea cycle
PWY−7211: superpathway of pyrimidine deoxyribonucleotides de novo biosynthesis
PWY−5941: glycogen degradation II (eukaryotic)
PWY−6895: superpathway of thiamin diphosphate biosynthesis II
PWY−5505: L−glutamate and L−glutamine biosynthesis
Weight
d
Top 5 Metabolites within the Factor
Remission
No Remission
isocitrate
GlcNAc sulfate conjugate of C21H34O2 steroid**
montelukast
nicotinamide adenine dinucleotide (NAD+)
umbelliferone sulfate
3−dehydrocholate
chenodeoxycholate
catechol sulfate
7−ketolithocholate
7−ketodeoxycholate
2'−deoxyguanosine
2'−deoxycytidine
palmitoyl dihydrosphingomyelin (d18:0/16:0)*
N−palmitoyl−sphingosine (d18:1/16:0)
N−palmitoyl−sphinganine (d18:0/16:0)
synephrine
cholic acid glucuronide
skatol
(15:0)−anacardic acid
montelukast
tryptamine
3−formylindole
3−methyl−2−oxobutyrate
N−succinyl−leucine
phenethylamine
Weight
e
Performance
Training AUC
Test AUC
Logistic Regression
Random Forest
f
EoT, R²=0.02 , p=0.25 , n=43
Axis.1 [17.7%]
Axis.2 [8.9%]
D+R+
D+R−
D−R−
g
No Remission
Remission
-L-citrulline biosynthesis
-Urea cycle
-L-histidine degradation III
-L-histidine degradation I
-Anaerobic energy metabolism
-Anhydromuropeptides recycling
-Pyrimidine deoxyribonucleotides bio. III
-Gluconeogenesis III
-Adenosylcobalamin salvage I
-Flavin biosynthesis I
-Flavin biosynthesis III
-Gluconeogenesis I
-L-serine and glycine biosynthesis I
-Seleno-amino acid biosynthesis
-Phosphatidvlglycerol biosynthesis II
-Phosphatidvlglycerol biosynthesis I
-Gamma;-glutamyl cycle
Log2 Fold Change
h
Log2 FC (cpm)
-Bacteroides uniformis
-Bacteroides dorei
-Parabacteroides distasonis
-Bacteroides caccae
-Bacteroides uniformis CAG_3
-Bacteroides xylanisolvens
-Ruminococcus torques
-Bacteroides ovatus
-Alistipes finegoldii
-Bacteroides thetaiotaomicron
-Bacteroides vulgatus

Supplementary Figure 3. a, MOFA2 analyses, variance explained by each omics datasets; metabolomics and metagenomics. **b,** Seven MOFA2 factors were identified, five of which (Factors 1-5) were significantly different between POIT outcome groups. Boxplots show the median (centre line), 25th and 75th percentiles (box bounds), and whiskers extend to values within 1.5× the interquartile range. Statistical comparisons were performed using two Wilcoxon rank-sum test. **c,** Top five microbial pathways contributing to weight of each factor. **d,** Top five metabolites contributing to weight of each factor. Orange and blue lollipop colors represent negative and positive effect on factor weight, respectively. **e,** Comparison of average AUC between logistic regression and random forest models in predicting remission outcome based on five metabolites from Factor 2. Boxplots show the median (centre line), 25th and 75th percentiles (box bounds). **f,** Fecal metabolite composition is not different between POIT outcome groups at the end of avoidance (Euclidean distance matrix. $n=43$, $R^2 = 0.02$; $P = 0.25$). **g,** Gut microbial pathways enriched in microbiome of children who developed remission (blue bars) versus no remission (orange bars). Two-sided linear mix-effect model ($P<0.05$, $P.FDR>0.05$). **h,** The *ptpA* gene is mostly encoded by *Bacteroides* species including *B. dorei, B. uniformis, B. caccei,* and *B. xylanisolvens.* Log2 FC (CPM) represents the Log2 fold change of copies per million between remission and no-remission groups. Blue color represents an increased Log2 FC (cpm) in the no-remission group. Source data are provided as a Source Data file.
