# Supplementary material for: Gut microbial bile and amino acid metabolism associate with peanut oral immunotherapy failure
Source: Nat Commun. 2025 Jul 9;16:6330. doi: 10.1038/s41467-025-61161-x (PMC12241578; doi:10.1038/s41467-025-61161-x)
Supplement: Supplementary file 2 — Description of Additional Supplementary Files [file 41467_2025_61161_MOESM2_ESM.docx]

**Description of Additional Supplementary Files:**

**Supplementary Data 1.** Number of samples in microbiome analyses.

**Supplementary Data 2.** Patient Characteristics.

**Supplementary Data 3.** Pairwise beta-diversity comparisons between POIT-outcome groups and placebo arm (two-sided linear mix-effect model).

**Supplementary Data 4.** Variables that explain variance in 16S rRNA Sequencing-based fecal microbiota composition at each study time point (two-sided *PERMANOVA*).

**Supplementary Data 5.** Generalized Linear-Mix Model Results Applied to 16S rRNA data. Related to Figure 1G and 1H (two-sided linear mix-effect models).

**Supplementary Data 6.** Variables that explain variance in fecal metabolome at study time points (two-sided PERMANOVA).

**Supplementary Data 7**. Untargeted Metabolomic Modules (UMMs)

Supplementary Data 8. Metabolite modules that significantly distinguished POIT outcome groups (two-sided ANOVA, adjusted for participant age).

**Supplementary Data 9.** Variables that explain variance in shotgun metagenome sequencing-based fecal microbiome composition at each study time point (two-sided *PERMANOVA* based on Canberra distance matrix)

**Supplementary Data 10**. Bile acid genes found in metagenomics dataset

**Supplementary Data 11**. Differential abundance analyses on bile acid genes found in metagenomics dataset (two-sided linear mix-effect models).

**Supplementary Data 12**. Differential abundance analyses on bile acid genes at the taxonomic level (two-sided linear mix-effect models).

**Supplementary Data 13**. Area Under Curve (AUC) data from logistic regression (*glmnet*) and random forest (rf) machine learning models.

**Supplementary Data 14**. Microbial pathways that exhibit significant difference in relative abundance between remission and no remission groups (two-sided linear mix-effect models).

**Supplementary Data 15**. Microbial enzymes that exhibit significant difference in relative abundance between remission and no remission groups (two-sided linear mix-effect models).

**Supplementary Data 16**. Two-sided Pearson correlations between the copy number of *ptpA* and metabolomics modules
