# Supplementary material for: Gut microbial bile and amino acid metabolism associate with peanut oral immunotherapy failure
Source: Nat Commun. 2025 Jul 9;16:6330. doi: 10.1038/s41467-025-61161-x (PMC12241578; doi:10.1038/s41467-025-61161-x)
Supplement: Supplementary file 4 — Reporting Summary [file 41467_2025_61161_MOESM4_ESM.pdf]

nature portfolio

Corresponding author(s): Susan Lynch

Last updated by author(s): May 23, 2025

Reporting Summary

Nature Portfolio wishes to improve the reproducibility of the work that we publish. This form provides structure for consistency and transparency in reporting. For further information on Nature Portfolio policies, see our Editorial Policies and the Editorial Policy Checklist.

Statistics

For all statistical analyses, confirm that the following items are present in the figure legend, table legend, main text, or Methods section.

n/a	Confirmed	
☐	☒	The exact sample size (*n*) for each experimental group/condition, given as a discrete number and unit of measurement
☐	☒	A statement on whether measurements were taken from distinct samples or whether the same sample was measured repeatedly
☐	☒	The statistical test(s) used AND whether they are one- or two-sided<br>*Only common tests should be described solely by name; describe more complex techniques in the Methods section.*
☐	☒	A description of all covariates tested
☐	☒	A description of any assumptions or corrections, such as tests of normality and adjustment for multiple comparisons
☐	☒	A full description of the statistical parameters including central tendency (e.g. means) or other basic estimates (e.g. regression coefficient) AND variation (e.g. standard deviation) or associated estimates of uncertainty (e.g. confidence intervals)
☐	☒	For null hypothesis testing, the test statistic (e.g. *F*, *t*, *r*) with confidence intervals, effect sizes, degrees of freedom and *P* value noted<br>*Give P values as exact values whenever suitable.*
☐	☒	For Bayesian analysis, information on the choice of priors and Markov chain Monte Carlo settings
☐	☒	For hierarchical and complex designs, identification of the appropriate level for tests and full reporting of outcomes
☐	☒	Estimates of effect sizes (e.g. Cohen's *d*, Pearson's *r*), indicating how they were calculated

Our web collection on statistics for biologists contains articles on many of the points above.

Software and code

Policy information about availability of computer code

Data collection	16SrRNA and shotgun metagenome sequencing data were generated in-house and untargeted metabolomics data was generated by Metabolon Inc. See Methods section for further details. Clinical data including serum IgE levels and demographics were obtained from the Immune Tolerance Network and were previously published in the original study (Jones et al. 2022, Lancet).
Data analysis	Generalized linear mix-effect models were employed on longitudinal microbiome data to determine differences in microbial taxa, microbial pathways, metabolites between POIT outcome groups (D+R+, D+R-, D-R-) and remission outcome (Yes or No), using a custom script (https://github.com/lynchlab-ucsf/lab-code/blob/master/SigTaxa/ManyModelScript.R) that employs multiple statistical models (Linear Model, Compound Poisson Linear Model, Poisson, Negative Binomial, and Tweedie) and compared using the AIC before reporting the final estimate and p value. False-discovery corrections were made using the Benjamini-Hochberg method.<br><br>All custom R scripts including Many Model (Generalized linear mix-effect model) and PERMANOVA scripts used for data analyses and described in the Methods are available at https://github.com/lynchlab-ucsf. All R codes used to generate figures in this study will be made available from the corresponding author upon reasonable request.

For manuscripts utilizing custom algorithms or software that are central to the research but not yet described in published literature, software must be made available to editors and reviewers. We strongly encourage code deposition in a community repository (e.g. GitHub). See the Nature Portfolio guidelines for submitting code & software for further information.

Data

Policy information about availability of data

All manuscripts must include a data availability statement. This statement should provide the following information, where applicable:

- Accession codes, unique identifiers, or web links for publicly available datasets
- A description of any restrictions on data availability
- For clinical datasets or third party data, please ensure that the statement adheres to our policy

The untargeted metabolomics, shotgun metagenomics and amplicon sequencing data generated for this study will be deposited in the NCBI SRA database. Additional data will be made available upon request.

Research involving human participants, their data, or biological material

Policy information about studies with human participants or human data. See also policy information about sex, gender (identity/presentation), and sexual orientation and race, ethnicity and racism.

Reporting on sex and gender	Sex and gender information for the participants included in this study is provided in Table S2. No sex- or gender-specific analyses were performed.
Reporting on race, ethnicity, or other socially relevant groupings	Not applicable. Race and ethnicity data were not received and collected in this study.
Population characteristics	The median participant age was 3.2 (1.1-4.0) which is reported in Table s2.
Recruitment	This study is a secondary analysis of a previous clinical trial and no specific recruitment was performed for this study. Full details of the IMPACT clinical trial (NCT01867671), including the recruitment process, have previously been described in the Lancet paper by Jones et al 2022.
Ethics oversight	This study was approved by the Office of Human Research Ethics (OHRE), University of North Carolina, Chapel Hill on April 9, 2013. The parent study titled, "IMPACT: Oral Immunotherapy (OIT) for Induction of Tolerance and Desensitization in Peanut-Allergy was a randomized, double-blind, placebo-controlled, multi-center study comparing peanut oral immunotherapy (OIT) to placebo. Informed consent was obtained from a parent or guardian of all participants.

Note that full information on the approval of the study protocol must also be provided in the manuscript.

Field-specific reporting

Please select the one below that is the best fit for your research. If you are not sure, read the appropriate sections before making your selection.

- [x] Life sciences
- [ ] Behavioural & social sciences
- [ ] Ecological, evolutionary & environmental sciences

For a reference copy of the document with all sections, see nature.com/documents/nr-reporting-summary-flat.pdf

Life sciences study design

All studies must disclose on these points even when the disclosure is negative.

Sample size

Stool samples were collected by participants at home and stored at clinical collection sites at -80 °C. Of the 146 participants enrolled in the IMPACT clinical trial (intention-to-treat group), 93 completed the treatment through the avoidance phase (per-protocol group). A total of 388 fecal samples were collected from 144 participants. Among the 93 participants who completed the treatment (per-protocol group), 327 fecal samples were obtained from 90 participants - 245 from the POIT group and 82 from the placebo group (57 and 23 participants, respectively, Supplementary Table 1). Three per-protocol participants did not provide fecal samples at any time points. One per-protocol participant who did not develop POIT-induced desensitization but developed remission (D-R+) was excluded (5 fecal samples from 5 time points) from all data analyses as a single sample was insufficient for statistical analyses.

To maintain blinding, investigators did not have access to participant data until after 16S rRNA sequencing was completed and locked. As a result, all 388 fecal samples underwent 16S rRNA sequencing. High-quality 16S rRNA sequencing data were successfully generated for only 263 fecal samples from 79 participants, as some samples failed due to insufficient DNA, failed PCR, or did not pass the quality filtering and rarefaction (35,000 reads/samples). For analyses assessing the relationship between fecal microbiota composition and POIT outcomes, only samples from the per-protocol participants were included. However, baseline analyses, such as those presented in Fig. 1H, 1I, 1J, incorporated samples from all participants. In these cases, POIT outcomes were not a consideration, as the goal was to evaluate correlations between baseline bacterial phylogenetic diversity and serum IgE levels in peanut-allergic children.

For shotgun metagenomics, we focused on three key time points (baseline, end of treatment, and end of avoidance) while excluding mid-maintenance and end-of-build-up samples. This decision was based on cost considerations and the significant associations observed between fecal microbiota and different clinical outcomes at these three time points (Supplementary Table 4). DNA extracted for 16S rRNA sequencing with at least 100 ng of remaining material from these three time points was used for shotgun metagenome sequencing. Placebo participants who provided samples at baseline but did not provide fecal samples at the other two key time points (8 participants, Supplementary Fig. 1A) were excluded because we observed no significant differences in baseline fecal bacterial composition and diversity between the POIT and

	Placebo groups. High-quality shotgun metagenomics data were obtained from 80 participants (184 samples).<br><br>Finally, metabolomics data were generated for 58 participants who had corresponding shotgun metagenomics data and sufficient remaining material from all three key time points. Two participants were excluded because their samples were fully utilized during DNA extraction. Additionally, 20 participants who provided only baseline samples without subsequent time points were excluded to enable longitudinal metagenomics and metabolome integrative analyses with matching patient IDs. Thus, we retained data for 58 participants, 22 fewer than the number analyzed for shotgun metagenomics (Supplementary Fig. 1A, & Supplementary Table 1).
Data exclusions	No data was excluded.
Replication	Because we used fecal samples collected at different time points during a clinical trial, 16S rRNA sequencing, shotgun metagenomic sequencing, and untargeted metabolomics experiment did not include replication as a standard procedure. In vitro experiments reported in Figure 4i include 18 participants (biological replicates) from two time points.
Randomization	The method for randomization of participants into treatment and placebo groups was previously described in the original study. No further randomization was performed in this study because the clinical trial was completed at the time of initiation of this study.
Blinding	The investigators of this study were blinded and did not have access to the metadata until the initial generation of the 16S rRNA data.

Reporting for specific materials, systems and methods

We require information from authors about some types of materials, experimental systems and methods used in many studies. Here, indicate whether each material, system or method listed is relevant to your study. If you are not sure if a list item applies to your research, read the appropriate section before selecting a response.

Materials & experimental systems

n/a	Involved in the study
☒	☐ Antibodies
☒	☐ Eukaryotic cell lines
☒	☐ Palaeontology and archaeology
☒	☐ Animals and other organisms
☐	☒ Clinical data
☒	☐ Dual use research of concern
☒	☐ Plants

Methods

n/a	Involved in the study
☒	☐ ChIP-seq
☒	☐ Flow cytometry
☒	☐ MRI-based neuroimaging

Clinical data

Policy information about clinical studies

All manuscripts should comply with the ICMJE guidelines for publication of clinical research and a completed CONSORT checklist must be included with all submissions.

Clinical trial registration	NCT01867671
Study protocol	Full details of the IMPACT clinical trial (NCT01867671) have been previously published (Jones et al. 2022)
Data collection	Full details of the IMPACT clinical trial (NCT01867671) have been previously published (Jones et al. 2022)
Outcomes	Full details of the IMPACT clinical trial (NCT01867671) have been previously published (Jones et al. 2022). Briefly, at baseline, 146 peanut-allergic children were randomized (2:1) to either POIT or placebo treatment. After a dose escalation phase of 30-weeks, children in the POIT arm received 2,000 mg peanut protein (lightly roasted, partly defatted [12% fat]) while the placebo group received oat flour for 104 weeks (total blinded treatment period 134 weeks). Participants who passed the 5-g peanut protein, double-blind, placebo-controlled, food challenge (DBPCFC) at the end of treatment (week 134) were categorized as desensitized (D+). Independent of the DBPCFC outcome at week 134, all participants avoided peanut consumption for 24 weeks (avoidance period), and those who passed the 5-g peanut protein DBPCFC at the end of this avoidance period (week 160) were categorized as being in remission (R+).

Plants

Seed stocks	not applicable
Novel plant genotypes	not applicable
Authentication	not applicable
